# New Insights into Human Milk Oligosaccharide Profiles in China: Findings from a Large-Scale Analysis of Human Milk

**DOI:** 10.3390/nu18030417

**Published:** 2026-01-27

**Authors:** Shuang Liu, Qisijing Liu, Che Pan, Sinéad T. Morrin, Rachael H. Buck, Xiang Li, Yingyi Mao, Shuo Wang

**Affiliations:** 1 Key Laboratory of Special Diet Nutrition and Health Research of China National Light Industry Council, School of Medicine, Nankai University, Tianjin 300071, China; 2 Research Institute of Public Health, Nankai University, Tianjin 300071, China; 3 Abbott Nutrition Research & Development Center, Shanghai 200233, China; 4 Abbott Nutrition Research & Development Center, Columbus, OH 43215, USArachaelbuck64@gmail.com (R.H.B.); 5 Abbott Nutrition Research & Development Center, Singapore 138668, Singapore

**Keywords:** human milk oligosaccharides, phenotypes, breast milk, lactation

## Abstract

**Background/Objectives:** This study systematically analyzed the concentration dynamics of human milk oligosaccharides (HMOs) and the distribution characteristics of secretory (Se) and Lewis (Le) phenotypes in China. **Methods:** A total of 1462 breast milk samples were collected from lactating mothers in six major regions of China, including Changchun, Lanzhou, Chengdu, Tianjin, Guangzhou, and Shanghai. We quantified 17 major HMOs by high-performance anion exchange chromatography-pulsed amperometric detection (HPAEC-PAD), and Se/Le phenotypes were determined to evaluate regional differences and distribution patterns. **Results**: Total HMO concentration in breast milk showed a significant downward trend within 200 days postpartum and stabilized after 200 to 400 days. Fucosylated HMOs accounted for the highest proportion 60.0–83.0%, among which 2′-FL had the largest concentration 903.4–2832.7 mg/L; acetylated HMOs 8.4–17.6% and sialylated HMOs 8.2–25.3% accounted for relatively lower proportions. This study further divided breast milk into four phenotypes based on HMO characteristics: 72.49% of the samples were Se+/Le+, 6.145% were Se+/Le−, 20.12% were Se−/Le+, and 1.24% were double negative (Se−/Le−). Se+ and Le+ phenotypes accounted for 78.7% and 92.6% of the total population, respectively. The total concentration of HMOs in breast milk of different phenotypes was significantly different, with the average total HMO concentration of Se+/Le+ breast milk being the highest (8342 mg/L), while that of Se−/Le− breast milk being the lowest (4532 mg/L). Se+ phenotype was associated with higher levels of fucosylated HMOs, including 2′-fucosyllactose (2′-FL) and lacto-*N*-fucopentaose I (LNFP I), and lower levels of lacto-*N*-tetraose (LNT) and sialyl-lacto-*N*-tetraose b (LST b) compared to other phenotypes. Most HMOs reached their highest concentrations during the colostrum (CM) and transitional milk (TM) stages, followed by a progressive decline with lactation, with phenotype-specific variations evident across all HMOs. Notably, certain HMOs, such as 3-FL, 3′-SL, DFL, and LNDFH II, exhibited distinct temporal patterns. **Conclusions**: This study revealed the Se/Le phenotype distribution and dynamic characteristics of HMOs in the Chinese mother-infant population, offering a valuable reference for global breast milk composition databases and infant nutrition research.

## 1. Introduction

Human breast milk is universally recognized as the optimal nutritional source for infants. In alignment with World Health Organization (WHO) guidelines, exclusive breastfeeding is strongly advised for the first six months of life, with continued breastfeeding alongside complementary foods recommended until at least two years of age. Beyond its foundational role in nourishment, breast milk confers critical immunological protection and is associated with both immediate and lifelong health benefits. These include reduced risks of gastrointestinal infections, acute illnesses, and chronic conditions such as type 2 diabetes and obesity [1,2,3,4,5]. Emerging evidence also underscores its positive correlation with enhanced cognitive development and improved socioemotional outcomes [6,7,8]. The intricate composition of human milk extends beyond macronutrients (e.g., lactose, lipids) and micronutrients, encompassing bioactive molecules with multifunctional roles. A prime example is human milk oligosaccharides (HMOs), which rank as the third most abundant solid constituent, following lactose and lipid, present at concentrations of 20–25 g/L in colostrum and declining to 5–15 g/L in mature milk [9,10,11]. Characterized by structural diversity and functional versatility, HMOs are now recognized not only as prebiotics fostering beneficial gut microbiota but also as mediators of pathogen inhibition and immune modulation in preclinical studies [12]. Recent advances in analytical techniques have further elucidated their complexity of structures, as well as the nutritional and protective mechanisms, solidifying their importance in infant health and development [13,14].

HMOs function as prebiotics, playing a pivotal role in shaping the infant gut microbiota. Accumulating evidence confirms their ability to promote colonization of beneficial bacteria, particularly Bifidobacteria, which dominate the intestinal ecosystem of breastfed infants, constituting over 50–70% of the total microbial population [15]. This selective advantage arises from Bifidobacteria’s unique genetic machinery, which encodes specialized oligosaccharide transporters and glycoside hydrolases to efficiently metabolize HMOs, thereby enhancing their survival and proliferation [16]. Beyond microbiota modulation, preclinical studies also suggested that HMOs serve as a frontline defense against pathogens by disrupting bacterial adhesion and inhibiting growth kinetics of diverse enteric pathogens, thereby protecting infants from invasive infections. Notably, the majority of HMOs undergo microbial fermentation in the colon, producing metabolites such as short-chain fatty acids (SCFAs) and aromatic lactic acids with localized and systemic immunomodulatory effects. A small portion of HMOs are also directly absorbed intact into systemic circulation of both breastfed infants and adults, where they directly influence host physiology through mechanisms such as immune regulation and enhancement of epithelial barrier function. Emerging research further reveals that HMOs and their derivatives, including sialic acid, extend their benefits to neurodevelopment [17,18]. Clinical studies suggest that early exposure to the most prevalent HMOs, namely 2′-fucosyllactose (2′-FL), correlates positively with cognitive development in breastfed infants, while metabolites such as sialic acid facilitate neuronal signaling, synaptogenesis, and brain maturation [17,19]. These multifunctional roles underscore the significance of HMOs not only in gut health but also in systemic development and long-term neurological outcomes, including improved cognitive performance and language development during infancy, as well as higher intelligence quotient (IQ) scores and a reduced risk of childhood behavioral disorders during childhood [19,20,21].

HMOs are synthesized in the mammary gland through the action of glucosyltransferases, forming structures composed of 3–23 monosaccharide units [22,23]. These oligosaccharides are derived from a lactose core, Gal β1-4 Glc, which is extended through the addition of lacto-*N*-biose (Gal β1-3 GlcNAc, β1-3 linkage) or *N*-acetyllactosamine (Gal β1-4 GlcNAc, β1-6 linkage). The structural diversity of HMOs arises from further modifications involving five key monosaccharides: D-glucose (Glc), D-galactose (Gal), *N*-acetylglucosamine (GlcNAc), L-fucose (Fuc), and *N*-acetylneuraminic acid (Neu5Ac, or sialic acid) [24,25]. Fucosylation or sialylation of the core structures defines the classification of HMOs. Fucosylated variants (45–83% of total HMOs) are formed via α1-2, α1-3, or α1-4 glycosidic bonds, while sialylated variants (6–21%) result from α2-3 or α2-6 linkages. Acetylated variants (6–35%) represent an additional structural category terminating in *N*-acetylglucosamine [26,27,28,29,30,31]. To date, over 200 distinct HMO structures have been identified in human milk, with their biological effects directly linked to their unique isomeric configurations. This structural complexity underscores the functional versatility of HMOs, as subtle variations in glycosidic bonds or terminal modifications can significantly alter their interactions with microbial and host receptors. Consequently, elucidating HMO profiles provides critical insights into their role in infant health and development.

HMOs exhibit a conserved structural framework, yet their profiles display marked interindividual variation in both composition and concentration. Studies have shown that HMO concentrations in breast milk fluctuate with lactational stage [32,33]. In addition to the lactational stage, maternal genetics and environmental factors such as diet, physiology, and geographic location significantly influence HMO concentration and composition [30,34,35]. The maternal genetic phenotype contributes to HMO profiles. The variation in HMO profiles is largely determined by two key genetic factors: the Secretor (Se) and Lewis (Le) phenotypes, which are governed by the maternal galactoside 2-α-l-fucosyltransferase 2 (FUT2) and galactoside 3/4-l-fucosyltransferase (FUT3) genes on chromosome 19 [22,36]. Secretor-positive (Se+) mothers, who express FUT2, predominantly produce HMOs with α1-2 linkages, such as 2′-FL and lacto-*N*-fucopentaose I (LNFP I) [37]. In contrast, Lewis-positive (Le+) mothers, who express FUT3, predominantly produce HMOs with α1-3/4 linkages, such as 3-FL and lacto-*N*-fucopentaose II (LNFP II) [37]. Additionally, Lewis antigen expression is jointly regulated by FUT2 and FUT3, with distinct Lewis determinants arising from variations in galactose linkage and fucosylation position [38].

The Se+/Le+ phenotype exhibits the highest compositional diversity, encompassing 2′-FL, 3-FL, difucosyllactose (DFL), lacto-*N*-tetraose (LNT), lacto-*N*-neo-tetraose (LNnT), lacto-*N*-fucopentaose I/II (LNFP I/2), and lacto-*N*-difucohexaose I/II (LDFH 1/2). In contrast, the Se−/Le+ group lacks α1-2-fucosylated HMOs, with its profile comprising 3-FL, LNT, LNnT, LNFP II, and LDFH III. The Se+/Le− phenotype exhibits intermediate diversity, featuring 2′-FL, LNT, LNnT, and LNFP I. Finally, the rare Se−/Le− group, characterized by absent FUT2 and FUT3 activity, shows minimal fucosylation [28,39]. Secretor phenotypes further modulate total HMO concentrations, with non-secretors displaying significantly lower levels than their secretor counterparts [33,40]. Emerging research now prioritizes comparative analyses of HMO composition and abundance across these four groups, refining our understanding of genetic determinants in HMO biosynthesis.

Beyond genetic determinants, environmental factors, including geography, delivery season, maternal diet, age, ethnicity, parity, and delivery mode, may influence the concentration and composition of HMOs. A multinational study of 410 lactating women across 11 cohorts revealed marked geographical disparities: Swedish mothers exhibited higher levels of 3-FL and LNFP III compared to African mothers, while Peruvian mothers demonstrated elevated 2′-FL, 3-FL, and 3′-SL concentrations, with 98% of this cohort classified as secretors [30]. Notably, ethnically similar populations residing in distinct regions displayed divergent HMO profiles, particularly in LNT and DSLNT levels.

Further comparisons reveal geographic variations in HMO compositions. Asian mothers showing higher siallylacto-*N*-tetraose c (LST c) concentrations than Caucasian counterparts, Hispanic populations exhibiting lower siallylacto-*N*-tetraose b (LST b), and Chinese and South African mothers producing more LNFP III than Finnish mothers, alongside reduced 2′-FL and LNFP I levels [30,37,41]. A large contributor to HMO compositional differences between Chinese mothers is thought to be due to maternal phenotypic groups Se+/Le+,Se−/Le+,Se+/Le−,Se−/Le−. For instance, 2′-FL abundance strongly correlates with Se+/Le+ and Se+/Le− phenotypes, whereas elevated 3-FL typically indicates Se−/Le+ phenotypes. Seasonal and perinatal factors may influence HMO level and composition. Gambian mothers produce less LNnT during wet seasons [42], while preterm delivery was associated with elevated 3′-sialyllactose (3′-SL), 6′-sialyllactose (6′-SL), LNT, and LNDFH I compared to term delivery [43]. Despite these insights, research on drivers of HMO variability in Chinese populations remains limited, underscoring the need for expanded studies in diverse regional populations.

Previous studies have measured HMO profiles in Chinese populations [33,44,45,46], predominantly focused on early lactation stages (≤2 months postpartum), with limited longitudinal data extending to 12 months [47]. Critical gaps remain in understanding how HMO composition evolves across lactation periods and geographic regions in China, particularly when stratified by maternal Se/Le phenotypes. To address this, we conducted a multicenter longitudinal study within the Chinese Breast Milk Cohort, analyzing 1462 breast milk samples from six cities over 13 months postpartum. This investigation aimed to quantify temporal changes in HMO concentrations during the first year of lactation, correlate Se/Le phenotypic groups with distinct HMO profiles, examine the impact of geographic variation on HMO diversity, and explore associations among HMO components in relation to maternal and infant characteristics. By integrating maternal, genetic, and environmental factors, our findings elucidate the dynamic interplay shaping HMO composition and provide novel insights into phenotype-driven and region-specific variations in Chinese breast milk.

## 2. Materials and Methods

### 2.1. Study Design and Participants

**Figure 1:**
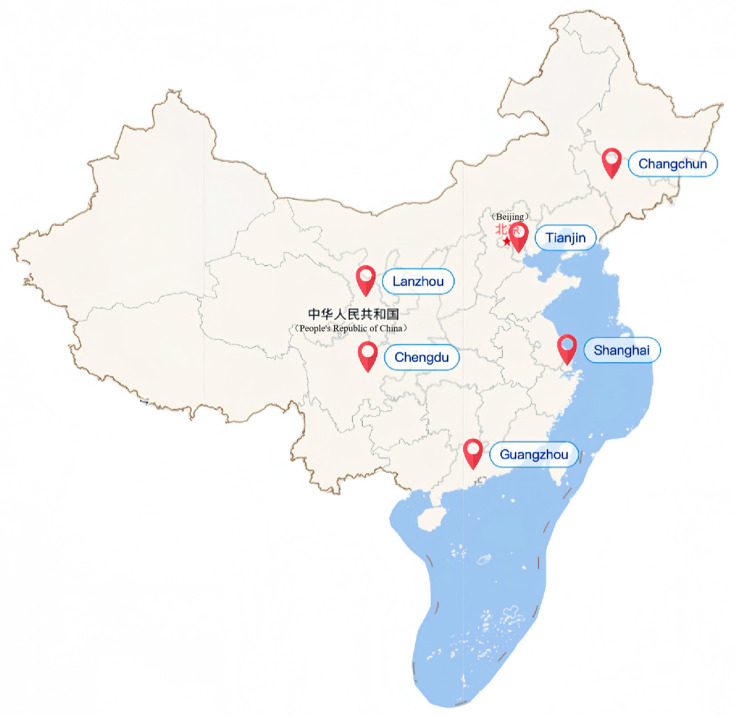
Multicenter maternal nutrition and infant investigation study location.

This study was conducted within the framework of the Maternal Nutrition and Infant Investigation (MUAI), a longitudinal cohort initiated in 2017 to examine maternal and infant health in China. With institutional support from regional hospitals, approximately 2000 mother-infant pairs were recruited across 6 geographically diverse cities: Tianjin (Northern China), Changchun (Northeast China), Lanzhou (Northwest China), Chengdu (Southwest China), Guangzhou (South China), and Shanghai (East China) (Figure 1). These regions were selected to capture variations in dietary practices and lifestyle patterns representative of the Chinese population.

Eligibility criteria included mothers aged 20–35 years who had resided in their respective regions for ≥2 years, breastfed for >3 months, delivered full-term singletons (37–42 weeks of gestation), and had infants with Apgar scores > 8. Exclusion criteria encompassed diagnosed maternal or infant chronic/acute illnesses, infectious diseases, or the use of medications that could affect nutritional metabolism. Breast milk samples were collected from the 6 regional cohorts. The study protocol adhered to the Declaration of Helsinki and was approved by the Ethics Committee of Tianjin hospital of ITCWM Nankai hospital (NKYY_YX-IRB 2018_005_01, 6 February 2018, Tianjin, China). Written informed consent was obtained from all participants, and the trial was registered with the China Clinical Trial Center (ChiCTR1800015387, 1 March 2018).

### 2.2. Breast Milk Sample Collection

Breast milk samples were collected at five lactation stages: colostrum (CM, 0–5 days postpartum), transitional milk (TM, 10–15 days postpartum), mature milk stage 1 (M1, 40–45 days postpartum), mature milk stage 2 (M2, 200–240 days postpartum), and mature milk stage 3 (M3, 300–400 days postpartum). A standardized collection protocol was implemented across all 6 study sites. Samples were obtained between 8:00 and 11:00 a.m. using manual or electric pumps to fully empty one breast. The thoroughly mixed milk sample was then divided: a portion was allocated for human milk research, while the remainder was returned to the mother for infant feeding. Considering the variation in milk volume across different lactation stages, the collected volumes in the MUAI study were standardized as follows: 5–10 mL for colostrum, 10–15 mL for transitional milk, and 30–50 mL for mature milk. Collected samples were immediately placed in insulated containers for transport to local laboratories. If delayed transport occurred, samples were temporarily stored at −20 °C and transferred within 24 h. Upon arrival, samples underwent controlled thawing in a 25 °C water bath for 20 min, followed by vortexing (2 min) to ensure homogeneity. Visually homogeneous samples (free of oil layers or particulate matter) were aliquoted and archived at −80 °C for subsequent analysis.

### 2.3. HMOs Analysis

For colostrum and transitional milk, an aliquot of 100 μL of homogenized sample was diluted 10-fold with lab water (type 1, 18.2 MΩ·cm), vortexed for 1 min to achieve homogeneity, and filtered through a 0.22 μm nylon membrane to deplete proteins and lipids. Mature milk samples (an aliquot of 200 μL) underwent identical processing, including dilution, vortexing, and filtration. All filtrates were subsequently transferred to injection vials for chromatographic analysis.

The identification and quantification of native HMOs were performed using a Thermo Fisher ICS 5000 (Thermo, Waltham, MA, USA) series high-performance anion-exchange chromatography system with pulsed amperometric detection (HPAEC-PAD). The system comprised a ternary gradient pump and an electrochemical cell operating under a carbohydrates quad potential waveform, with an Ag/AgCl reference electrode. Four chromatographic methods were used to analyze 17 HMOs to achieve separation of all isomers. For 2′-FL, 3-FL, LNT, and LNnT, chromatographic separation was performed on a separation column (CarboPac™ PA1, 4 × 150 mm, Thermo, Waltham, MA, USA) connected to a guard column (CarboPac™ PA1, 4 × 50 mm, Thermo, Waltham, MA, USA), with the column temperature maintained at 25 °C. The injection mode was full-loop injection, and the flow rate was 1.0 mL/min. The elution gradient was as follows: 0.00–12.00 min, 60 mM NaOH; 12.00–20.00 min, 60–155 mM NaOH; 20.00–30.00 min, 155 mM NaOH; 30.01–43.00 min, 125 mM NaOH/18 mM NaOAc; 43.01–48.00 min, 100 mM NaOH/240 mM NaOAc; 48.01–63.00 min, 60 mM NaOH. Ion chromatography instrument parameters were used to separate DFL, LNFP I, LNFP II, LNFP III, and LNDFH I. The sugar column guard column PA1 was connected in series with the sugar analysis column PA1. The column temperature was 25 °C. The injection mode was full-loop injection, and the flow rate was 1.0 mL/min. The elution gradient was as follows: 0.00–12.00 min, 60 mM NaOH; 12.00–26.00 min, 130 mM NaOH; 26.01–31.00 min, 100 mM NaOH/240 mM NaOAc; 31.01–41.00 min, 60 mM NaOH.

Ion chromatography instrument parameters were used to separate LNDFH 2 and TFLNH. The sugar column guard column PA1 was connected in series with 2 sugar analysis columns PA1. The column temperature was 28 °C, the injection mode was full-loop injection, and the flow rate was 0.6 mL/min. The elution gradient was as follows: 0.00–14.00 min, 140 mM NaOH; 14.01–26.00 min, 100 mM NaOH/240 mM NaOAc; 26.01–41.00 min, 140 mM NaOH. Ion chromatography instrument parameters were used to separate 3′-SL, 6′-SL, LST a, LST b, LST c, and DSLNT. The sugar column guard column PA1 was connected in series with the sugar analysis column PA1. The column temperature was 25 °C, the injection mode was full-loop injection, and the flow rate was 1.0 mL/min. The elution gradient was as follows: 0.00–16.00 min, 100 mM NaOH/60 mM NaOAc; 16.00–40.00 min, 100 mM NaOH/60–210 mM NaOAc; 40.01–44.00 min, 100 mM NaOH/240 mM NaOAc; 44.01–54.00 min, 100 mM NaOH/60 mM NaOAc.

### 2.4. Classification of Maternal Phenotype as Secretor and Lewis

The maternal phenotypes—defined by the expression of two specific fucosyltransferases, namely FUT2 and FUT3—were determined following previously published literature [44,48]. Maternal phenotypes are categorized into secretor phenotypes and Lewis phenotypes. Secretor phenotypes were classified based on the α1-2 concentration in breast milk. Lewis phenotypes were classified based on the α1-4 concentration in breast milk. The Se/Le type of breast milk was classified according to the content of 2′-FL, DFL, LNFP I, LNFP II, LNDFH I, and LNDFH 2 in breast milk as reported in previous studies.

### 2.5. Statistical Analysis

All exploratory and descriptive statistical analyses were performed with the use of SPSS 25.0 (IBM SPSS Corp., Armonk, NY, USA) and GraphPad Prism 9.5.1 (LLC of Boston, MA, USA). HMO concentrations were reported as the median (25–75th percentile) since the data presented a non-normal distribution. The minimum sample size was calculated by the formula (z × SD/E)^2^). E was the average value of HMO concentration multiplied by the allowable error of the method. SD was the standard deviation of HMO concentrations, and z = 1.96. The variation of HMO concentrations among 6 geographical sites was explored with the independent nonparametric test (Kruskal–Wallis One-way ANOVA, all pairwise, adjust *p*-value). The proportions of secretors and non-secretors were compared by the Chi-square test. All statistical analyses were considered significant at *p* < 0.05 (two-sided). The sum of the concentrations of 2′-FL, 3-FL, LNFP I, LNFP II, LNFP III, LNDFH I, LNDFH 2, TFLNH, LNT, LNnT, 3′-SL, 6′-SL, LST a, LST b, LST c, and DSLNT represented the total HMO concentrations.

## 3. Results

### 3.1. Maternal and Infant Characteristics Across Study Sites

A total of 1462 healthy mothers participated in the study, including 244 from Changchun, 299 from Lanzhou, 281 from Chengdu, 229 from Tianjin, 342 from Guangzhou, and 363 from Shanghai. Overall, the average (SD) age at delivery of the participant mothers was 29.9 (3.5) years. The pre-pregnant body mass index (BMI) and pre-delivery BMI of the participant mothers were 21.1 (2.9) kg/m^2^ and 26.6 (3.3) kg/m^2^, respectively. The weight gain during pregnancy was 14.6 (5.6) kg. The gestational age of the participant mothers was 39.2 (1.6) weeks. The rate of vaginal delivery and primipara was 59% and 71%, respectively. 49% of infants were female. The infant birthweight and birth length were 3397.6 (637.9) g and 49.9 (2.1) cm, respectively. The characteristics of participant mothers at each city are listed in Table 1. Except for the maternal age at delivery and the infant’s sex, the characteristics of mothers and infants showed significant differences across cities.

### 3.2. The Proportion of Secretor and Lewis Phenotypes Across Lactation Stages and Cities

**Figure 2:**
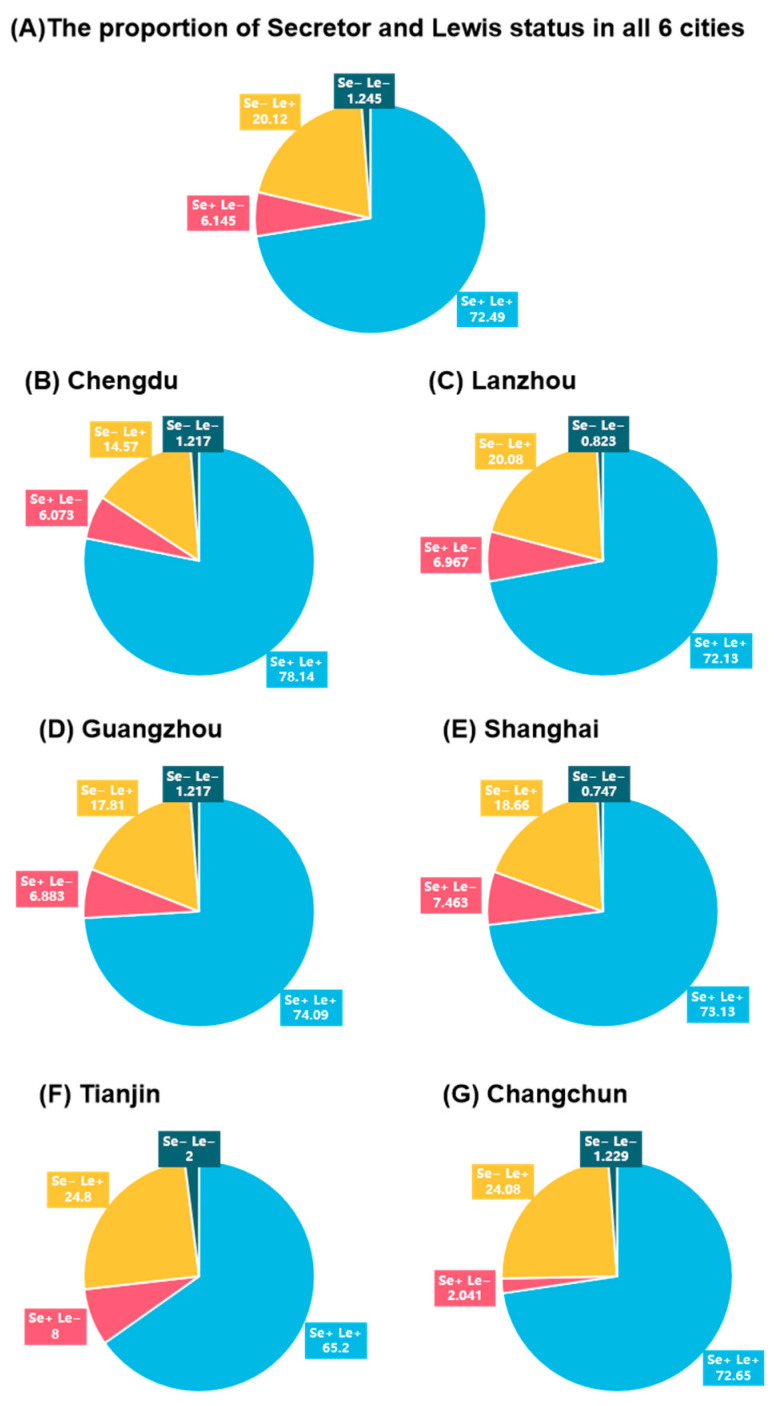
The proportion of secretor and Lewis phenotypes in Chinese milk at 0–400 postpartum periods of each city. (**A**) The proportion of Secretor and Lewis status in all 6 cities, (**B**) The proportion of Secretor and Lewis status in Chengdu, (**C**) The proportion of Secretor and Lewis status in Lanzhou, (**D**) The proportion of Secretor and Lewis status in Guangzhou, (**E**) The proportion of Secretor and Lewis status in Shanghai, (**F**) The proportion of Secretor and Lewis status in Tianjin, (**G**) The proportion of Secretor and Lewis status in Changchun.

As shown in Figure 2A, the Se+/Le+ phenotype was the most prevalent across all lactation stages, accounting for 72.49% of the overall population. The Se−/Le+ phenotype was the second most common, representing 20.12% of the population. In contrast, the Se+/Le− and Se−/Le− phenotypes were relatively rare, consistently comprising ~10% of the population across all lactation stages.

The distribution of secretor and Lewis phenotypes in the six cities aligns with that of the total population. Nevertheless, notable geographical variations in the distribution of these phenotypes existed (Figure 2B–G). The most common phenotype across all cities was Se+/Le+, with the highest prevalence in Chengdu (78.14%) and the lowest in Tianjin (65.20%). The second most frequent phenotype was Se−/Le+ (20.1%), particularly common in Changchun (24.08%) and Tianjin (24.8%), indicating more non-secretor mothers in those regions. Se+/Le− and Se−/Le− phenotypes were rare, making up 6.145% and 1.244% of the total population, respectively. Notably, Tianjin had higher rates of Se− and Le− phenotypes (2%), likely due to regional genetic, ethnic, or environmental influences (Table 2).

Overall, secretor-positive (Se+) mothers represented 78.7% of the cohort, while Lewis-positive (Le+) mothers accounted for 92.6%, indicating that most Chinese lactating women possess the enzymatic capacity to synthesize fucosylated HMOs (Table 2). Despite this, regional variation in the secretion phenotypes was not significant. Chi-square analysis revealed no significant difference in the proportion of secretor and Lewis phenotypes among different cities, although there was a significant intercity difference in the secretion phenotype alone (chi-square *p* = 0.03).

### 3.3. Total Human Milk Oligosaccharides Content Across Secretor and Lewis Phenotypes

**Figure 3:**
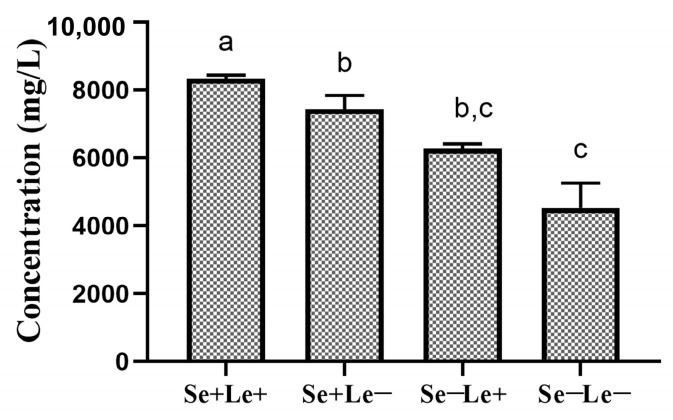
Contents of a total of 17 oligosaccharides in the milk of secretor and Lewis phenotypes (mg/L) at 0–400 postpartum periods. Note: ^a,b,c^ Values within a row in individual cities with different superscript letters were significantly different (adjusted *p* < 0.05 by the Dunn correction for multiple tests) according to an independent nonparametric test (Kruskal-Wallis one-way ANOVA, all pairwise).

As shown in Table 3, the average of total contents of 17 HMOs in Se+/Le+, Se+/Le−, Se−/Le+, and Se−/Le− types of breast milk is 8342 mg/L, 7449 mg/L, 6276 mg/L, and 4532 mg/L, respectively. There are significant differences in the total HMOs content in the four types of breast milk (*p* < 0.001). The results are shown in Figure 3.

### 3.4. Contents of Fucosylated, Acetylated, and Sialylated Oligosaccharides in Breast Milk Across Secretor and Lewis Phenotypes

**Figure 4:**
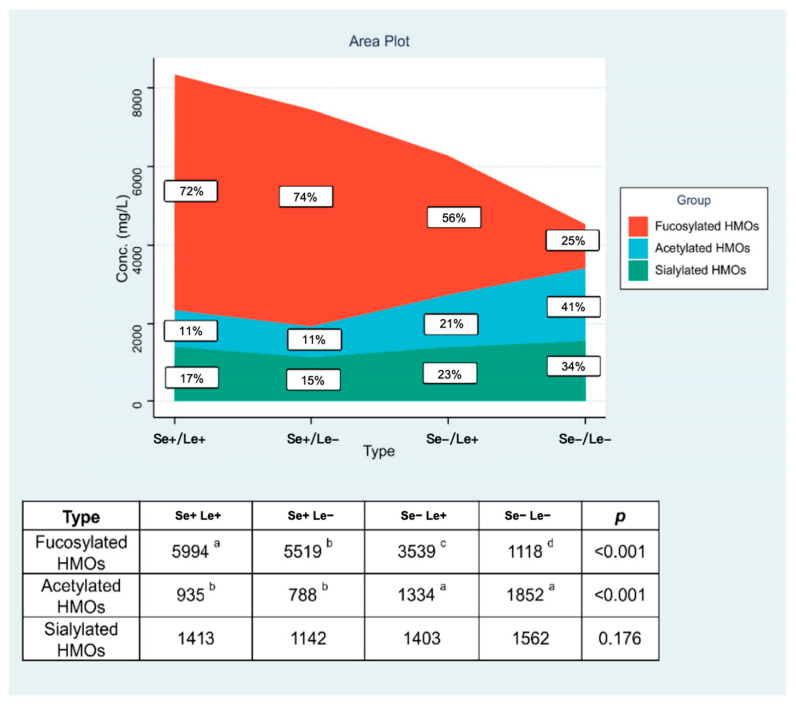
Contents of the three major types of oligosaccharides in the milk of secretor and Lewis phenotypes at 0–400 postpartum periods (*n* = 1462, MEAN, mg/L). Note: ^a,b,c,d^ Values within a row in individual cities with different superscript letters were significantly different (adjusted *p* < 0.05 by the Dunn correction for multiple tests) according to an independent nonparametric test (Kruskal–Wallis one-way ANOVA, all pairwise).

As demonstrated in Figure 4, the Se/Le phenotype exerted a pronounced influence on the composition of fucosylated and acetylated HMOs (*p* < 0.001), while sialylated HMOs remained statistically consistent across all four phenotypic categories throughout the 400-day postpartum period.

### 3.5. Distribution of 17 HMOs in Breast Milk Across Secretor and Lewis Phenotypes

**Figure 5:**
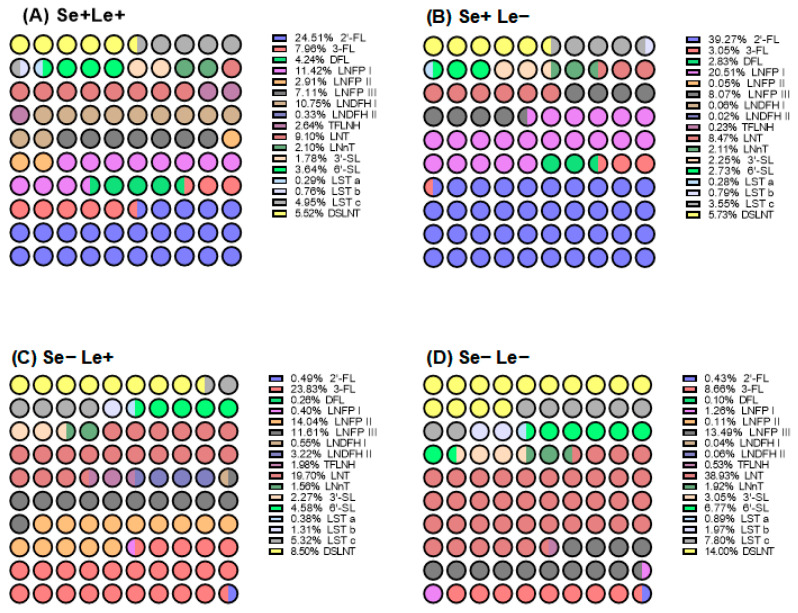
The proportion of 17 HMOs in the milk of secretor and Lewis phenotypes of all study participants.

As shown in Figure 5, the distribution of HMOs is strongly influenced by maternal Se/Le phenotypes: Se+/Le+ and Se+/Le− milk is dominated by secretor-dependent HMOs such as 2′-FL (24.51% vs. 39.27%), LNFP I (11.42% vs. 20.51%), and LNDFH I level (10.75% vs. 0.06%). Se−/Le+ milk shows elevated Lewis-dependent HMOs, such as 3-FL (23.83%), LNT (19.70%), and LNFP II (14.04%). In contrast, Se−/Le− milk lacks both antigens and is characterized by high proportions of core structures such as LNT (38.93%), DSLNT (14.00%), and LNFP III (13.49%).

**Figure 6:**
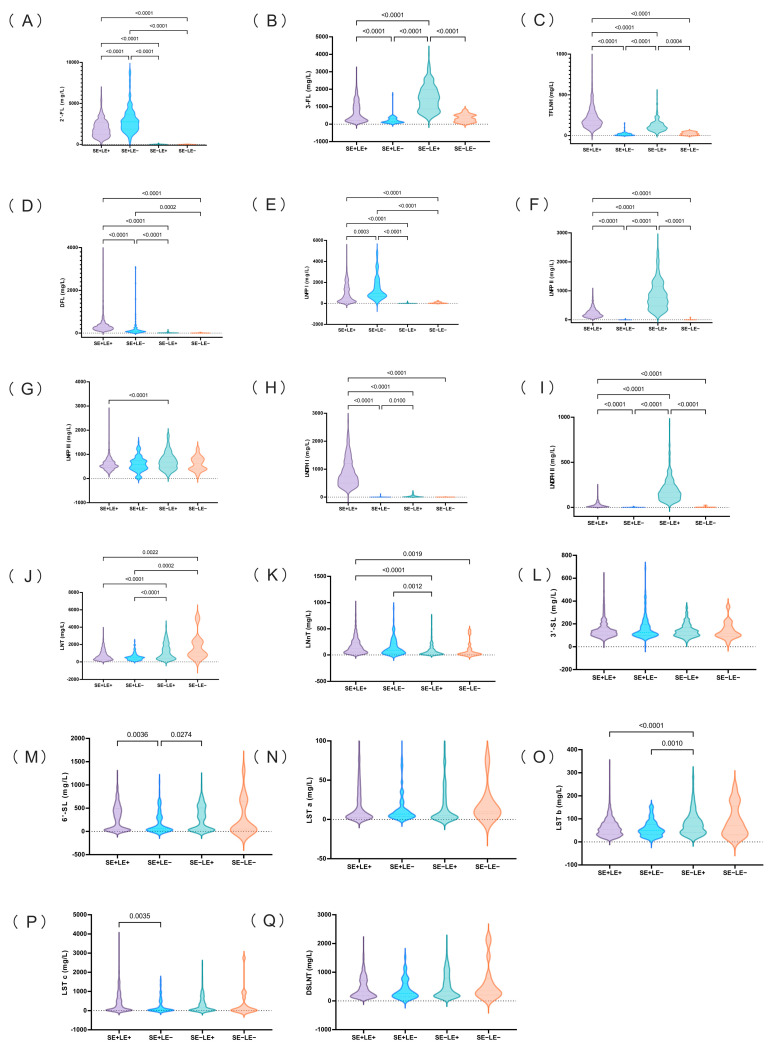
Contents of 17 HMOs in the milk of secretor and Lewis phenotypes at 0–400 postpartum periods (MEAN, mg/L). Note: Statistical significance was determined using the Kruskal–Wallis one-way ANOVA followed by Dunn’s post-hoc pairwise comparisons (adjusted *p* < 0.05). (**A**) 2′-FL, (**B**) 3-FL, (**C**) TFLNH, (**D**) DFL, (**E**) LNFP I, (**F**) LNFP II, (**G**) LNFP III, (**H**) LNDFH I, (**I**) LNDFH I, (**J**) LNT, (**K**) LNnT, (**L**) 3′-SL, (**M**) 6′-SL, (**N**) LST a, (**O**) LST b, (**P**) LST c, (**Q**) DSLNT.

With the exception of 3′-SL, LST, and DSLNT, the remaining 14 HMOs showed significant differences across the four types of breast milk (Figure 6). The contents of 2′-FL, DFL, and LNFP I in Se+ breast milk were significantly higher than those in Se− breast milk, and the contents of LNT were significantly lower than those in Se− breast milk. In addition, the content of DSLNT in Se+ breast milk is lower than that in Se− breast milk, but not significantly. The contents of 3-FL, TFLNH, LNFP II, LNDFH I, and LNDFH 2 in Le+ breast milk were significantly higher than those in Le− breast milk. Beyond the HMOs previously documented in the literature (2′-FL, DFL, LNFP I, LNFP II, LNDFH I, and LNDFH 2), our study further identified 3-FL and TFLNH as robust biomarkers for distinguishing Le+ from Le− phenotypes, whereas LNT and LNnT exhibited specificity in differentiating Se+ and Se− groups. Other HMOs demonstrated limited discriminative power in classifying the Se/Le phenotype.

### 3.6. Trends in the Changes of 17 HMOs in Breast Milk Across Secretor and Lewis Phenotypes

As shown in Table 4, the changing trends of 15 HMOs across three major milk phenotypes (Se+/Le+, Se+/Le−, and Se−Le+) exhibited broadly similar patterns across lactation. Due to the limited sample size of the Se−Le− group at each lactation stage (CM: *n* = 2, TM: *n* = 3, M1: *n* = 3, M2: *n* = 3, and M3: *n* = 8), this group was excluded from trend analysis.

In Se+/Le+ and Se+/Le− milk, 2′-FL levels declined significantly with lactation progression (*p* < 0.001), stabilizing between 200 and 400 days postpartum (M2–M3). Although the content of 2′-FL in Se−/Le+ milk was markedly lower, it followed a similar downward trend across lactation. Conversely, 3-FL showed a continuous increased trend in all three groups, with Se−Le+ milk presenting the highest levels (up to 2236 mg/L at M3). DFL followed a U-shaped curve, with elevated levels at early (CM) and late (M3) lactation stages across Se+/Le+ and Se+/Le− phenotypes. The content of LNFP I decreased throughout the lactation period and plateaued between 200 and 400 days postpartum. The content of LNFP II first increased and then decreased, with the highest content in transitional milk, and tended to be stable between 200 and 400 days postpartum. LNFP III exhibited a gradual decrease over time in Se+/Le+ and Se−Le+ milk (*p* < 0.001), with only marginally significant change observed in Se+/Le− milk (*p* = 0.051). LNDFH I concentrations significantly declined across lactation in both Se+/Le+ and Se−Le+ milk (*p* < 0.001), whereas LNDFH 2 showed a gradual increase in Se+/Le+ milk, from 24 mg/L in CM to 46 mg/L in M3 (*p* < 0.001). In Se−Le+ milk, LNDFH 2 levels remained high throughout lactation (~200 mg/L), but the content in Le-type breast milk is lower than 10 mg/L. The trend of TFLNH differed by phenotype: in Se+/Le+ milk, it increased from CM to TM, followed by a decrease; in Se−Le+ milk, it showed an initial decrease, then increased again from TM to M3 (*p* < 0.001). LNT levels showed an inverted U-shape in all phenotypes, peaking in TM (e.g., 2223 mg/L in Se−Le+ milk). Thereafter, the Se−Le+ phenotype exhibited a sustained decline from TM to M3, whereas Se+ phenotypes decreased after the peak but remained relatively stable between M2 and M3. LNnT declined over time in all three groups (Se+/Le+, Se+/Le−, Se−Le+) (*p* < 0.001) and tended to plateau between 200 and 400 days postpartum. In terms of 3′-SL, levels were highest in CM, followed by a sharp decline during the TM stage and a further reduction to lower steady levels in mature milk (M1 and M2). A slight rebound was observed at the M3 only in Se+/Le+ milk, but concentrations did not return to CM levels. LST a, LST b, and LST c all decreased consistently throughout lactation, especially LST c, which dropped from 914–1130 mg/L in CM to 15–26 mg/L by M3 in all phenotypes (*p* < 0.001). Lastly, DSLNT exhibited unique trends. In Se+/Le+ milk, DSLNT content dropped significantly in the first 240 days but rose again by 300–400 days postpartum (from 161 mg/L to 206 mg/L, *p* < 0.001), forming a late-stage recovery pattern. In contrast, in Se+/Le− milk, DSLNT declined from TM to M1 and remained relatively stable from M1 to M3. In Se−Le+ milk, DSLNT also declined significantly from TM to M2, after which levels stabilized between M2 and M3.

### 3.7. Correlations Among HMOs and Their Associations with Maternal and Infant Factors

**Figure 7:**
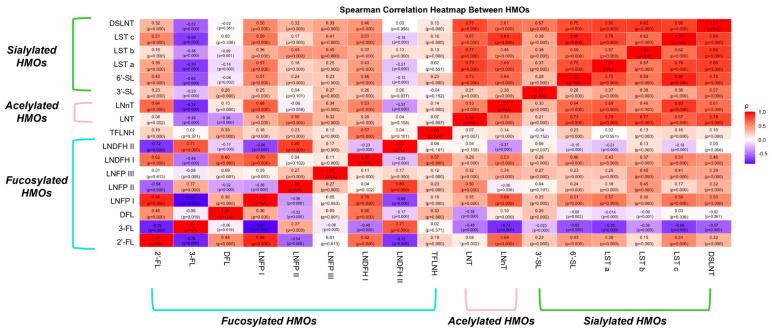
Correlation matrix of HMO types at 0–400 postpartum periods, with blue color indicating negative correlations and red color indicating positive correlations.

As shown in Figure 7, we calculated the correlations among various HMOs, categorizing them into three groups: fucosylated HMOs (blue bracket), acetylated HMOs (red bracket), and sialylated HMOs (green bracket). 2′-FL was positively associated with DFL, LNFP I, LNDFH I, and TFLNH, LNT, LNnT, and all sialylated HMOs, but negatively associated with 3-FL, LNFP II, and LNDFH II. 3-FL exhibited negative correlations with 2′-FL, DFL, LNFP I, LNFP III, and LNDFH I and all sialylated HMOs, but positively associated with LNFP II and LNDFH II. This may stem from substrate competition during the synthesis of fucosylated HMOs, particularly between (α1-2)- and (α1-3/4)-fucosylated HMOs.

In contrast, sialylated HMOs (e.g., 3′-SL, 6′-SL, LST a, LST b, LST c, and DSLNT) demonstrated strong positive correlations (r = 0.28–0.90), reflecting their structural similarities. Additionally, sialylated HMOs showed significant positive correlations with acetylated HMOs (e.g., LNT and LNnT) but exhibited significant negative or very weak positive correlations with fucosylated HMOs. This discrepancy may be linked to the presence of a shared Galβ1-4Glc structure, implying potential substrate competition among these HMOs.

**Figure 8:**
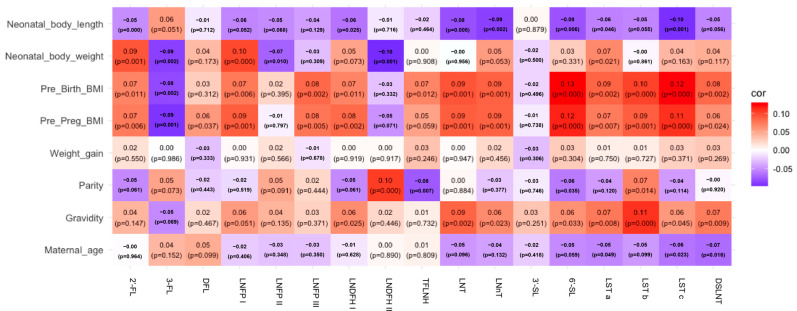
Correlation matrix of HMO types and maternal and infant factors at 0–400 postpartum periods, with blue color indicating negative correlations and red color indicating positive correlations.

We further analyzed the associations between maternal factors (age, gravidity, parity, gestational weight gain, pre-pregnancy, and pre-delivery BMI) and HMO concentrations over the first 400 days postpartum (Figure 8). Maternal age at delivery was negatively correlated with LST a, LST c, and DSLNT. Gravidity showed positive correlations with acetylated HMOs (LNT and LNnT) and most sialylated HMOs (6′-SL, LST a, LST b, LST c, and DSLNT). Parity was positively correlated with LNDFH 2 and LST b but negatively correlated with TFLNH, and 6′-SL. Maternal obesity also significantly influenced HMO levels. Pre-pregnancy BMI was negatively correlated with 3-FL but positively correlated with 2′-FL, DFL, LNFP I, LNFP III, LNDFH I, LNT, LNnT, and most sialylated HMOs (6′-SL, LST a, LST b, LST c, and DSLNT). Pre-delivery BMI had broader effects; it was positively correlated with 2′-FL, LNFP I, LNFP III, LNDFH I, TFLNH, LNT, LNnT, and most sialylated HMOs (6′-SL, LST a, LST b, LST c, and DSLNT), yet negatively correlated with 3-FL. Gestational weight gain showed no significant association with HMO levels.

Lastly, we examined the correlations between infant growth metrics and HMOs. Birth body length was negatively associated with LNDFH I, LNT, LNnT, 6′-SL, LST a, and LST c. Birth weight was positively correlated with 2′-FL, LNFP I, and LST a but negatively with 3-FL, LNFP II, and LNDFH 2.

Despite the weak correlation coefficients, these findings suggest that HMO composition and levels are influenced by multiple maternal and infant factors and may be closely linked to infant growth and development.

## 4. Discussion

In this large-scale, multicenter study, we comprehensively assessed the absolute concentrations of representative HMOs in 1462 breast milk samples from six geographically diverse Chinese cities. Additionally, we systematically examined how HMO composition varied across lactation stages, maternal Se/Le phenotypes, and geographic regions. By presenting detailed quantitative profiles up to 13 months postpartum, our findings provide a robust foundation for developing a Chinese HMO reference database and inform strategies to tailor infant nutrition based on maternal phenotype and lactation stage. Overall, the lactation stage emerged as the primary driver of HMO concentration changes, with total HMO levels and individual components exhibiting distinct, time-dependent patterns. The total concentration of 17 HMOs in human milk exhibited a declining trend from 0 to 200 days postpartum, followed by a stable concentration between 200 and 400 days. Similarly, the levels of fucosylated HMOs decreased during the 0–200-day period and remained stable thereafter from 200 to 400 days. Colostrum contained the highest median concentration of fucosylated HMOs, reaching 8 g/L. In contrast, non-fucosylated neutral HMOs showed an initial increase followed by a decrease over the first 400 days, peaking in transitional milk at 10–15 days with a median concentration of 1.8 g/L, and remained stable between 200 and 400 days. Sialylated HMOs displayed a decreasing trend from 0 to 200 days and stabilized thereafter up to 400 days. The highest median concentration of sialylated HMOs was observed in colostrum, at 2.8 g/L.

Since the Se and Le genotypes directly determine the activity of FUT2 and FUT3 enzymes that catalyze the fucosylation of HMOs, understanding their population distribution is essential for interpreting the observed HMO profiles in this cohort. Notably, while the lactation stage reflects the trajectory of HMO concentrations, whereas Se and Le phenotypes—determined by FUT2 and FUT3 activity—represent fundamental genetic determinants that shape inter-individual variability in HMO composition, particularly for fucosylated structures. We systematically examined the Se/Le phenotypes of mothers in various Chinese cities. In our cohort, the Se+ to Se− ratio (78.7%:21.4%) was consistent with the general distribution pattern reported in Finland (87%:13%) [49], Brazil (89%:11%) [48], Spain (66%:34%) [50], and Malaysia (88%:12%) [51]. Moreover, in our cohort, the proportion of Le+ to Le− mothers was 92.6%:7.4%, consistent with the general distribution pattern observed in previous studies in China and other populations. Similar distributions have been reported among Chinese mothers (91–94% Le+) [44] and in Germany (94%:6%), a multinational study (87%:13%) [47], and an international cohort of 371 mothers [52]. Of all Se/Le phenotypes, the most predominant phenotype of our study participants was Se+/Le+ (72.5%), followed by Se−/Le+ (20.1%) and Se+/Le− (6.1%), with Se−/Le− being rare (1.2%). In Asian populations, the prevalence of the Se+/Le+ phenotype varies between 55% and 73%, while Se−/Le+ ranges from 20% to 31%, Se+/Le− from 6% to 11%, and Se−/Le− from 3% to 5% [53,54,55,56]. These distributions differ notably from African populations, where the distributions are approximately 54% for Se+/Le+, 14% for Se−/Le+, 25% for Se+/Le−, and 7% for Se−/Le−, with the proportion of Se+/Le− individuals being notably higher than in Asian populations [57]. In our study, only two mothers exhibited the Se−/Le− phenotype, underscoring its rarity in Asian populations. Notably, no significant regional differences in Se/Le phenotype distributions were observed across the six Chinese cities, suggesting that maternal fucosyltransferase activity is primarily determined by genetic background rather than geographic or environmental factors. Together, these findings provide robust population-level data on Se/Le phenotype distribution among Chinese mothers and support their relevance for interpreting inter-individual variability in HMO profiles.

Several studies have noted significant differences in oligosaccharide profiles between different geographical locations and provinces [30,44,58]. By examining the impact of geographical location on maternal phenotype, we found significant differences in the proportions of Se+ and Se− among six Chinese provinces, but no significant difference in the proportions of Le+ and Le−. Overall, more southerly regions had a higher proportion of Se+ mothers. Various factors, such as dietary habits and seasonal variations, may be closely associated with the province the mothers come from, affecting HMO levels. More than 90% of the mother-infant pairs recruited in the six regions of this study were of Han ethnicity and presumably had similar cultural and dietary habits, as well as a similar genetic background, which may have led to regional similarities. Future research can delve deeper into this issue by collecting samples from a broader range of locations and performing multivariate analyses.

Our findings confirm that the total HMO concentrations and compositional profiles varied significantly by phenotype. Se+/Le+ milk exhibited the highest total HMO concentrations, averaging 8342 mg/L, followed by Se+/Le− 7449mg/L, Se−/Le+ 6276mg/L, and Se−/Le− 4532mg/L. Fucosylated HMOs accounted for the largest proportion 60.0−83.0% across all samples, while non-fucosylated acetylated HMOs 8.4−17.6% and sialylated HMOs 8.2−25.3% contributed to smaller proportions. The stage of lactation also had a significant impact on HMO levels. Total HMO concentrations decreased as postpartum time progressed, with the most pronounced decline occurring after the first 200 days (CM to M2). This trend is consistent with findings from other longitudinal studies [33,44]. For example, 2′-FL content in Se+/Le+ milk dropped from 3262 mg/L in early lactation to 1104 mg/L after 200 days. This trend is consistent with prior studies in other populations. LNFP I also showed a marked decline, while 3-FL increased steadily throughout lactation, especially between 200 and 400 days postpartum. This pattern suggests a distinct role for 3-FL in later infancy, potentially supporting ongoing gut maturation, immune modulation, and neurodevelopment. The observed increase in 3-FL is likely attributable to its biosynthesis via FUT3 (encoded by the Le gene). Indeed, Se−/Le+ milk exhibited the highest 3-FL concentrations, consistent with prior evidence that 3-FL predominates in Se−/Le+ individuals due to substrate competition. Our data also support earlier reports that maternal genetic variation strongly influences enzyme activity and HMO composition, with 3-FL, 2′-FL, and LNFP I serving as biomarkers for differentiating phenotypes.

This study found that breast milk 2′-FL levels averaged 3–4 g/L in the first 45 days postpartum and 1 g/L between 200 and 400 days of secretor mothers. Additionally, secretor mothers provide high 2′-FL levels for up to one year, supporting early infant development. Preclinical studies associate 2′-FL with improved microbiota, pathogen defense, anti-inflammatory effects, and cognitive development. Clinical studies show that supplementation of 2′-FL 0.2−1.0g/L to infant formula reduces plasma inflammatory cytokines and TNF-α by 29–83%, similar to levels in breastfed infants [13]. Another double-blind study reported that after 16 weeks, infants fed 2′-FL-supplemented formula 1.0g/L showed altered Bifidobacterium metabolism and increased activity of two HMO-related glycosyl hydrolase families [59]. Among secretor mothers, the second most abundant fucosylated HMO was LNFP I and was exclusively present in the Se+ participants. Its concentration showed a significant decrease throughout lactation, consistent with findings from other studies [49]. These findings suggest that high-level HMOs such as 2′-FL in breast milk may promote early infant development and health through gut health benefits, including improved microbiota, enhanced pathogen defense, and reduced inflammation.

This study also extends the focus to other fucosylated HMOs, particularly highlighting 3-FL as one of the most predominant fucosylated HMOs aside from 2′-FL. Notably, the concentration dynamics of 3-FL exhibit a distinct time-dependent pattern: during the first year postpartum, its levels demonstrate a consistent upward trend throughout lactation. Although the decline in 2′-FL concentration during lactation was not statistically significant, it aligns with trends reported in previous studies. The two isomers, 2′-FL and 3-FL, differ in the position of the fucose attachment on the lactose molecule: in 2′-FL, fucose is linked via an α1-2 glycosidic bond, while in 3-FL, it is linked via an α1-3 glycosidic bond. Both isomers play crucial roles in infant health, supporting gut microbiota development, immune regulation, antiviral and antimicrobial defense, and neurological maturation, as shown in preclinical and clinical studies [60,61]. 3-FL is one of the few HMOs known to increase significantly during lactation, suggesting its importance beyond the exclusive breastfeeding period. This oligosaccharide may provide benefits to infants aged 6 to 12 months, a period beyond the typical lactation phase [62]. The concentration of 3-FL increased across lactation in all groups except the Se−/Le− group, where a reduction at month three was followed by a recovery in month four The Se−/Le+ group showed the highest 3-FL levels. Synthesis of 3-FL is influenced by the expression of the Le gene, while FUT3 is the key enzyme responsible for its biosynthesis. Other studies have observed higher levels of 3-FL in non-secretor milk compared to secretor milk, suggesting that the availability of the enzyme substrates leads to competition, thereby influencing the relative levels of fucosylated HMOs [63,64]. Ning et al. (2024) [65] reported that mothers from the Han Hebei region exhibited higher 3-FL levels than the average values found in other Chinese cohorts. These differences were primarily attributed to the presence or absence of α1,2-fucosylated HMOs, such as 2′-FL, LNFP I, and LNDFH I, in secretor and non-secretor milk.

Among sialylated HMOs, 6′-SL, LST c, and DSLNT were the most abundant across all Se/Le groups. Overall, 6′-SL and LST c consistently declined with lactation, whereas 3′-SL showed a peak in CM, a sharp decline by the transitional stage, stabilization at low levels during mature milk, and a slight late-stage rebound limited to Se+/Le+ milk. DSLNT, another prominent sialylated HMO, showed a consistent decline across lactation stages, except in Se+/Le+ milk, where it slightly increased between 200 and 400 days postpartum. The dynamic changes in HMO concentrations during lactation and their complex relationships highlight the potential roles of specific HMOs in infant development. DSLNT exhibited a significant negative correlation with advancing lactation, in agreement with previously reported data [30]. The biological relevance of these compositional changes warrants further investigation.

Maternal characteristics also correlated with HMO concentrations. Maternal age showed negative correlations with LST a, LST c, and DSLNT, suggesting that older mothers may produce lower levels of certain sialylated HMOs, potentially impacting functions related to infant neurodevelopment and immunity [66]. Pre-pregnancy and pre-delivery BMI also demonstrated correlations with a wide range of HMOs, highlighting the potential importance of maternal metabolic status during late pregnancy and lactation. Although most correlations were weak in magnitude, the consistent patterns underscore the multifactorial regulation of HMO biosynthesis by maternal physiology and its potential impact on infant growth. These observations highlight the importance of personalized nutritional strategies during lactation, particularly for populations with metabolic risk factors or multiple maternity histories and warrant further mechanistic studies to clarify causality and clinical significance.

While existing research on Chinese lactation has predominantly centered on early postpartum stages (<6 months), our investigation extends the methodological rigor to 13 months post-delivery, providing new insights into longitudinal HMO trajectory evolution. Despite its strengths, including a nationally representative cohort n=1462 and HPAEC-PAD-based quantification of 17 principal HMOs, this study acknowledges several constraints. Notably, while our analysis encompassed key fucosylated, sialylated, and acetylated structures, the targeted HMOs represent <10% of the documented structural diversity in human milk (>200 identified variants), although they represent over 80% of the quantified HMOs in human milk [67]. Additionally, although spanning six geographical regions, demographic homogeneity persists (the ethnicity of >90% of participants was Han), necessitating validation in multi-ethnic cohorts with distinct genetic backgrounds and dietary patterns. Crucially, the absence of infant biometric linkage data leaves the biological relevance of observed HMO fluctuations unresolved, highlighting an opportunity for subsequent investigations to establish clinical correlations between temporal HMO profiles and pediatric developmental endpoints.

## 5. Conclusions

This study establishes the first large-scale, longitudinal profile of human milk oligosaccharides (HMOs) in China, encompassing diverse lactation stages (0–400 days postpartum) and six major geographic regions. Our data reveal that HMO composition is modulated by maternal secretor/Le phenotypes and dynamic lactational timelines. These findings provide insights for feeding strategies that integrate maternal genotyping and stage-specific milk biochemistry. By creating the most comprehensive Chinese HMO reference database to date (1462 samples), this work fills a critical gap in global lactational biology while providing an evidence base for developing tailored formula milk and optimizing breastfeeding guidelines. Future translational research could prioritize linking these HMO signatures to infant gut microbiota maturation, immune competency metrics, and neurodevelopmental outcomes.

## Figures and Tables

**Table 1:** Characteristics of participant mothers at each city (MEAN ± SD or %).

Characteristic	Changchun (*n* = 244)	Lanzhou (*n* = 299)	Chengdu (*n* = 281)	Tianjin (*n* = 229)	Guangzhou (*n* = 342)	Shanghai (*n* = 363)	*p*
***Maternal***							
Age at delivery, years	29.9 ± 3.0	29.8 ± 4.3	29.5 ± 3.3	30.3 ± 3.2	29.7 ± 3.7	30.1 ± 3.0	0.054
Pre-pregnancy BMI, kg/m^2^	21.3 ± 3.0 ^a,b^	21.3 ± 2.8 ^a,b^	20.7 ± 2.5 ^b^	21.8 ± 3.4 ^a^	20.3 ± 2.5 ^b^	21.2 ± 2.8 ^a,b^	<0.001
Pre-delivery BMI, kg/m^2^	27.4 ± 3.8 ^a^	26.8 ± 3.0 ^a,b^	26.2 ± 2.8 ^b^	27.3 ± 3.4 ^a^	25.5 ± 3.1 ^b^	26.7 ± 3.6 ^a,b^	<0.001
Gestational weight gain, kg	16.5 ± 5.5 ^a^	14.3 ± 5.3 ^b^	14.2 ± 4.6 ^b^	14.6 ± 7.6 ^b^	13.5 ± 4.9 ^b^	14.7 ± 5.5 ^b^	<0.001
Gestational age, weeks	39.0 ± 1.1 ^b^	39.0 ± 2.1 ^a,b^	39.5 ± 1.6 ^a^	39.2 ± 1.7 ^a,b^	38.9 ± 1.4 ^b^	39.6 ± 1.0 ^a^	<0.001
Vaginal delivery, %	42% ^a^	59% ^b^	43% ^a^	68% ^b,c^	77% ^c^	61% ^b^	<0.001
Primipara, %	95% ^a^	50% ^b^	73% ^c,d^	77% ^d^	64% ^c^	74% ^c,d^	<0.001
***Infant***							
Birthweight, g	3380.1 ± 401.1 ^a,b^	3277.8 ± 445.1 ^b^	3409.5 ± 624.7 ^a,b^	3396.7 ± 490.8 ^a^	3542.6 ± 1004.9 ^a,b^	3332.3 ± 368.2 ^a,b^	0.025
Body length, cm	50.5 ± 1.7 ^a^	49.0 ± 2.5 ^c^	49.8 ± 1.5 ^b,c^	50.4 ± 2.6 ^a,b^	50.3 ± 2.3 ^b^	49.4 ± 1.3 ^c^	<0.001
Gender (Female %)	49%	47%	43%	51%	48%	53%	0.159

Note: ^a,b,c,d^ values within a row in individual cities with unlike superscript letters were significantly different (*p* < 0.05). Nonparametric test—Kruskal–Wallis one-way ANOVA (multiple comparisons: all pairwise, adj-*p*). Chi-square test (z-test—compare column proportions—adjust *p*-values (Dunn method)).

**Table 2:** Distribution of secretor+/− and Lewis+/− phenotypes among lactating women across six Chinese cities (%).

	Chengdu (*n* = 281)	Lanzhou (*n* = 299)	Guangzhou (*n* = 342)	Shanghai (*n* = 363)	Tianjin (*n* = 229)	Changchun (*n* = 244)	Total
**Se+**	84.21%	79.10%	81.07%	74.70%	80.59%	73.20%	78.7%
**Se−**	15.79%	20.90%	18.93%	25.30%	19.41%	26.80%	21.4%
**Le+**	92.67%	92.21%	91.90%	96.73%	91.80%	90.00%	92.6%
**Le−**	7.33%	7.79%	8.10%	3.27%	8.20%	10.00%	7.4%

**Table 3:** Contents of a total of 17 oligosaccharides in the milk of secretor and Lewis phenotypes (mg/L).

Se/Le Phenotypes	Average	SD	SE	*p* 25	*p* 50	*p* 75
Se+/Le+	8342	3327	102	5375	7543	11,093
Se+/Le−	7449	3706	393	4583	5855	10,582
Se−/Le+	6276	2368	141	4479	5741	8031
Se−/Le−	4532	3179	729	2357	3186	6199

Note: SD: standard deviation; SE: standard error; *p* 25: the 25th percentile; *p* 50: the 50th percentile; *p* 75: the 75th percentile.

**Table 4:** Change trends of 17 HMOs in the milk of secretor and Lewis phenotypes throughout lactation (mean, mg/L).

**2′-FL**		**CM**	**TM**	**M** **1**	**M2**	**M3**	***p***
	**Se+/Le+**	3262 ^a^	2433 ^b^	2151 ^c^	1238 ^d^	1104 ^d^	<0.001
	**Se+/Le−**	4948 ^a^	3580 ^a^	2628 ^a,b^	2164 ^b^	2313 ^b^	<0.001
	**Se−/Le+**	63 ^a^	39 ^a,b^	29 ^b^	13 ^c^	14 ^c^	<0.001
	**Se−/Le−**	24	24	28	15	15	-
**3-FL**		**CM**	**TM**	**M** **1**	**M2**	**M3**	*p*
	**Se+/Le+**	222 ^c^	243 ^c^	461 ^b^	1199 ^a^	1242 ^a^	<0.001
	**Se+/Le−**	127 ^b,c^	89 ^b,c^	182 ^b^	250 ^a,b^	363 ^a^	<0.001
	**Se−/Le+**	830 ^c^	941 ^c^	1400 ^b^	1946 ^a^	2236 ^a^	<0.001
	**Se−/Le−**	85	139	642	364	481	-
**DFL**		**CM**	**TM**	**M** **1**	**M2**	**M3**	***p***
	**Se+/Le+**	502 ^a^	222 ^b^	228 ^b^	346 ^a^	494 ^a^	<0.001
	**Se+/Le−**	255 ^a^	91 ^a,b^	61 ^b^	207 ^a,b^	326 ^a,b^	0.015
	**Se−/Le+**	30 ^a^	11 ^b,c^	16 ^a,b^	10 ^b,c^	15 ^b^	<0.001
	**Se−/Le−**	4	2	2	4	7	-
**LNFP I**		**CM**	**TM**	**M** **1**	**M2**	**M3**	***p***
	**Se+/Le+**	1936 ^a^	1619 ^a^	746 ^b^	214 ^c^	230 ^c^	<0.001
	**Se+/Le−**	3223 ^a^	2798 ^a^	1303 ^a,b^	729 ^b^	814 ^b^	<0.001
	**Se−/Le+**	54 ^a^	28 ^b^	44 ^a^	5 ^b^	3 ^b^	<0.001
	**Se−/Le−**	116	62	35	50	51	-
**LNFP II**		**CM**	**TM**	**M** **1**	**M2**	**M3**	***p***
	**Se+/Le+**	220 ^b^	336 ^a^	241 ^b^	197 ^b^	214 ^b^	<0.001
	**Se+/Le−**	3 ^b^	11 ^a^	7 ^a^	1 ^b^	1 ^b^	<0.001
	**Se−/Le+**	1111 ^a,b^	1365 ^a^	986 ^b^	525 ^c^	500 ^c^	<0.001
	**Se−/Le−**	1	0	1	29	1	-
**LNFP III**		**CM**	**TM**	**M** **1**	**M2**	**M3**	***p***
	**Se+/Le+**	780 ^a^	559 ^b,c^	561 ^b,c^	564 ^b^	505 ^c^	<0.001
	**Se+/Le−**	866	635	472	569	516	0.051
	**Se−/Le+**	1158 ^a^	782 ^b^	727 ^b^	557 ^c^	461 ^c^	<0.001
	**Se−/Le−**	907	590	687	408	594	-
**LNDFH I**		**CM**	**TM**	**M** **1**	**M2**	**M3**	***p***
	**Se+/Le+**	1338 ^a^	1250 ^a^	838 ^b^	493 ^c^	548 ^c^	<0.001
	**Se+/Le−**	3	3	10	2	6	0.055
	**Se−/Le+**	90 ^a^	38 ^b^	28 ^b^	9 ^c^	13 ^c^	<0.001
	**Se−/Le−**	1	1	0	1	3	-
**LNDFH II**		**CM**	**TM**	**M** **1**	**M2**	**M3**	***p***
	**Se+/Le+**	24 ^c^	17 ^d^	20 ^d^	31 ^b^	46 ^a^	<0.001
	**Se+/Le−**	5 ^a^	1 ^b^	1 ^a,b^	1 ^b^	1 ^b^	<0.001
	**Se−/Le+**	222 ^a,b^	223 ^a,b^	216 ^a,b^	151 ^b^	206 ^a^	0.020
	**Se−/Le−**	5	0	2	9	1	-
**TFLNH**		**CM**	**TM**	**M** **1**	**M2**	**M3**	***p***
	**Se+/Le+**	152 ^c^	316 ^a^	283 ^a^	155 ^b,c^	182 ^b^	<0.001
	**Se+/Le−**	14	18	12	17	22	0.451
	**Se−/Le+**	160 ^a^	95 ^c^	101 ^c^	117 ^b,c^	144 ^a,b^	<0.001
	**Se−/Le−**	17	16	14	17	35	-
**LNT**		**CM**	**TM**	**M** **1**	**M2**	**M3**	***p***
	**Se+/Le+**	910 ^b^	1459 ^a^	714 ^b^	323 ^c^	351 ^c^	<0.001
	**Se+/Le−**	945 ^a,b^	1249 ^a^	524 ^a,b^	401 ^b^	429 ^b^	<0.001
	**Se−/Le+**	2063 ^a^	2223 ^a^	1078 ^b^	513 ^c^	414 ^c^	<0.001
	**Se−/Le−**	3625	3668	1571	897	983	-
**LNnT**		**CM**	**TM**	**M** **1**	**M2**	**M3**	***p***
	**Se+/Le+**	375 ^a^	236 ^b^	143 ^c^	69 ^d^	54 ^d^	<0.001
	**Se+/Le−**	392 ^a^	233 ^a,b^	114 ^b^	82 ^b^	75 ^b^	<0.001
	**Se−/Le+**	277 ^a^	113 ^b^	64 ^b^	28 ^c^	16 ^c^	<0.001
	**Se−/Le−**	337	201	68	13	17	-
**3′-SL**		**CM**	**TM**	**M** **1**	**M2**	**M3**	***p***
	**Se+/Le+**	238 ^a^	143 ^b^	107 ^c^	115 ^c^	144 ^b^	<0.001
	**Se+/Le−**	282 ^a^	150 ^b^	92 ^b^	144 ^b^	170 ^b^	<0.001
	**Se−/Le+**	217 ^a^	148 ^b^	109 ^c^	106 ^c^	132 ^b,c^	<0.001
	**Se−/Le−**	288	151	99	108	121	-
**6′-SL**		**CM**	**TM**	**M** **1**	**M2**	**M3**	***p***
	**Se+/Le+**	469 ^b^	639 ^a^	312 ^c^	44 ^d^	32 ^e^	<0.001
	**Se+/Le−**	398 ^a^	601 ^a^	270 ^a^	37 ^b^	28 ^b^	<0.001
	**Se−/Le+**	464 ^a,b^	619 ^a^	328 ^b^	48 ^c^	27 ^c^	<0.001
	**Se−/Le−**	745	921	396	35	35	-
**LST a**		**CM**	**TM**	**M** **1**	**M2**	**M3**	***p***
	**Se+/Le+**	57 ^a^	47 ^a^	10 ^b^	3 ^c^	4 ^c^	<0.001
	**Se+/Le−**	59 ^a^	46 ^a^	6 ^b^	6 ^b^	7 ^b^	<0.001
	**Se−/Le+**	65 ^a^	43 ^a^	7 ^b^	3 ^c^	2 ^c^	<0.001
	**Se−/Le−**	148	99	27	7	9	-
**LST b**		**CM**	**TM**	**M** **1**	**M2**	**M3**	***p***
	**Se+/Le+**	84 ^a^	83 ^a^	69 ^b^	39 ^c^	41 ^c^	<0.001
	**Se+/Le−**	108 ^a^	80 ^a,b^	52 ^b^	42 ^b,c^	40 ^b,c^	<0.001
	**Se−/Le+**	136 ^a^	93 ^b^	92 ^b^	52 ^c^	46 ^c^	<0.001
	**Se−/Le−**	204	117	85	76	57	-
**LST c**		**CM**	**TM**	**M** **1**	**M2**	**M3**	***p***
	**Se+/Le+**	1130 ^a^	644 ^b^	241 ^c^	29 ^d^	26 ^d^	<0.001
	**Se+/Le−**	914 ^a^	605 ^a^	129 ^a^	21 ^b^	19 ^b^	<0.001
	**Se−/Le+**	932 ^a^	536 ^a^	198 ^b^	26 ^c^	15 ^c^	<0.001
	**Se−/Le−**	1827	761	205	10	16	-
**DSLNT**		**CM**	**TM**	**M** **1**	**M2**	**M3**	***p***
	**Se+/Le+**	852 ^a^	726 ^a^	353 ^b^	161 ^d^	206 ^c^	<0.001
	**Se+/Le−**	1018 ^a^	644 ^a,b^	255 ^b^	227 ^b,c^	254 ^b,c^	<0.001
	**Se−/Le+**	1080 ^a^	828 ^a^	418 ^b^	185 ^c^	199 ^c^	<0.001
	**Se−/Le−**	2131	1011	526	276	294	-

Note: ^a,b,c,d,e^ Values within a row in individual cities with different superscript letters were significantly different (adjusted *p* < 0.05 by the Dunn correction for multiple tests) according to an independent nonparametric test (Kruskal–Wallis one-way ANOVA, all pairwise); CM (colostrum, 0–5 days postpartum), TM (transitional milk, 10–15 days), M1 (mature milk stage 1, 40–45 days), M2 (mature milk stage 2, 200–240 days), and M3 (mature milk stage 3, 300–400 days).

## Data Availability

The datasets generated or analyzed during the current study are not publicly available due to the data management requirements of our institution but are available from the corresponding authors upon reasonable request.

## Abbreviations

The following abbreviations are used in this manuscript:

HMOs	human milk oligosaccharides
2′-FL	2′-fucosyllactose
3-FL	3-fucosyllactose
DFL	difucosyllactose
LNFP I	lacto-*N*-fucopentaose I
LNFP II	lacto-*N*-fucopentaose II
LNFP III	lacto-*N*-fucopentaose III
LNDFH I	lacto-*N*-difucohexaose I
LNDFH II	lacto-*N*-difucohexaose II
TFLNH	tri-fucosyl-lacto-*N*-hexaose
LNT	lacto-*N*-tetraose
LNnT	lacto-*N*-neo-tetraose
3′-SL	3′-sialyllactose
6′-SL	6′-sialyllactose
LST a/b/c	sialyl-lacto-*N*-tetraose a/b/c
DSLNT	disialyl-lacto-*N*-tetraose
SCFAs	short-chain fatty acids
Neu5Ac	*N*-acetylneuraminic acid
Glc	D-glucose
Gal	D-galactose
GlcNAc	*N*-acetylglucosamine
Fuc	L-fucose
Se	Secretor
Le	Lewis
Se+/Se−	Secretor-positive/negative
Le+/Le−	Lewis-positive/negative
FUT2	α1-2-fucosyltransferase gene
FUT3	α1-3-fucosyltransferase gene
CA19-9	Carbohydrate Antigen 19-9
HPAEC-PAD	high-performance anion exchange chromatography-pulsed amperometric detection
CM	colostrum
TM	transitional milk
M1	mature milk stage 1
M2	mature milk stage 2
M3	mature milk stage 3
ANOVA	Analysis of Variance
SPSS	Statistical Package for the Social Sciences
FDR	False Discovery Rate
SD	Standard Deviation
SE	Standard Error
WHO	World Health Organization
MUAI	Maternal Nutrition and Infant Investigation
ChiCTR	China Clinical Trial Center
TNF-α	Tumor Necrosis Factor-α
BMI	Body Mass Index
